# Selection and Assessment of Reference Genes for Quantitative PCR Normalization in Migratory Locust *Locusta migratoria* (Orthoptera: Acrididae)

**DOI:** 10.1371/journal.pone.0098164

**Published:** 2014-06-02

**Authors:** Qingpo Yang, Zhen Li, Jinjun Cao, Songdou Zhang, Huaijiang Zhang, Xiaoyun Wu, Qingwen Zhang, Xiaoxia Liu

**Affiliations:** 1 Department of Entomology, China Agricultural University, Beijing, China; 2 Department of Horticulture, Beijing Vocational College of Agriculture, Beijing, China; Uppsala University, Sweden

## Abstract

*Locusta migratoria* is a classic hemimetamorphosis insect and has caused widespread economic damage to crops as a migratory pest. Researches on the expression pattern of functional genes in *L. migratoria* have drawn focus in recent years, especially with the release of genome information. Real-time quantitative PCR is the most reproducible and sensitive approach for detecting transcript expression levels of target genes, but optimal internal standards are key factors for its accuracy and reliability. Therefore, it's necessary to provide a systematic stability assessment of internal control for well-performed tests of target gene expression profile. In this study, twelve candidate genes (*Ach*, *Act*, *Cht2*, *EF1α*, *RPL32*, *Hsp70*, *Tub*, *RP49*, *SDH*, *GAPDH*, *18S*, and *His*) were analyzed with four statistical methods: the delta Ct approach, geNorm, Bestkeeper and NormFinder. The results from these analyses aimed to choose the best suitable reference gene across different experimental situations for gene profile study in *L. migratoria*. The result demonstrated that for different developmental stages, *EF1α*, *Hsp70* and *RPL32* exhibited the most stable expression status for all samples; *EF1α* and *RPL32* were selected as the best reference genes for studies involving embryo and larvae stages, while *SDH* and *RP49* were identified for adult stage. The best-ranked reference genes across different tissues are *RPL32*, *Hsp70* and *RP49*. For abiotic treatments, the most appropriate genes we identified were as follows: *Act* and *SDH* for larvae subjected to different insecticides; *RPL32* and *Ach* for larvae exposed to different temperature treatments; and *Act* and *Ach* for larvae suffering from starvation. The present report should facilitate future researches on gene expression in *L. migratoria* with accessibly optimal reference genes under different experimental contexts.

## Introduction

In biological research, the fluorescence-based quantitative real-time reverse transcriptase PCR (qPCR) is the most reproducible and sensitive approach for gene expression analysis and has been widely used to measure and compare levels of gene transcription [1]–[3]. Although the technique of qPCR is usually described as the gold standard, the quality of results is influenced by several variables, including RNA stability, quantity, purity, reverse transcription efficiency and PCR efficiency [4], [5]. To avoid bias, a number of strategies have been proposed for normalization in previous studies [6], including sampling similar tissue weight or volume to ensure similar sample size [6], targeting genomic DNA [7] and using an ‘artificial’ RNA molecule [4]. The strategy based on sample size may be straightforward, but it may not be biologically representative because that different samples may not contain the same cellular material [6]. The method based on genomic DNA is rarely used, as the copy number per cell may vary and DNA is usually eliminated during the RNA extraction procedures. The normalization approach using an artificial molecule remains an unvalidated theoretical ideal. Therefore, the most suitable method for mRNA quantification is to include internal standards, which are mainly housekeeping genes.

The transcription levels of these widely used housekeeping genes, including *β-actin*, 18S ribosomal RNA (*18S rRNA*), elongation factor 1-α (*EF1α*) and glyceraldehyde-3-phosphate dehydrogenase (*GAPDH*) [8], [9] had been assumed to have uniform levels of expression that are unaffected by experimental conditions since these genes are necessary in fundamental cellular processes [10]. These genes have been used as single normalizers for many years. However, the expression of some commonly used reference genes could vary extensively and were unstable under a range of experimental conditions, which has been shown by several studies [11]–[18]. Furthermore, the mRNA transcript levels can differ from actual expression even up to 20-fold if the normalization gene is regulated by the experimental conditions [19]–[21]. Therefore, it's a key point to assure the expression of internal reference genes occur at a constant level. The endogenous control genes should be validated in different organism and for each specific experiment [10], [22]–[27].

The migratory locust *Locusta migratoria* is the most common locust subspecies that is widely distributed in eastern and southern Asia including China, Cambodia, Indonesia, Japan Thailand [28]. As a phytophagous insect, this pest feeds on gramineous and bulrush plants, and causes widespread economic damage to crops. Additionally, destructive outbreaks of locust is periodic [29]. In recent years, with the development of molecular technology and the release of the whole genome information of *L. migratoria*, qPCR has been widely used for study on phase changes [30] and gene expression in *L. migratoria* [31]. The systemic assessment of suitable internal control genes has been reported in several model insects such as *Bombyx mori* [32], *Apis mellifera* [33], [34], and *Tribolium castaneaum* [35]. For locusts, the initial several reference genes were validated for the brains of *Schistocerca gregaria* [36]. And then reference genes for locust were evaluated for *Chortoicetes terminifera* reared under different density treatments [37] and *L. migratoria* under hypobaric hypoxia stress [38]. There is no experimental data available on a systematic selection and assessment of reference genes in *L. migratoria* for gene profile analyses covering the commonly involved biotic and abiotic experimental contexts.

In this report, we analyzed the performance of twelve normalization genes (*Ach*, *Act*, *Hsp70*, *18S*, *EF1α*, *SDH*, *RPL32*, *His*, *Cht2*, *GAPDH*, *Tub*, and *RP49*) for *L. migratoria* in a set of biotic factors (embryo stage, larvae stage, adult stage and 13 tissues) and under three abiotic stresses (insecticide, temperature and starvation). This work will provide benefits for identifying normalization genes in future gene expression studies in *L. migratoria*, saving time and expense in selecting reference genes.

## Materials and Methods

### Ethics Statement

For this study, there were no specific permits being required for the insect collected. The eggs of *L. migratoria* were originally collected from Cangzhou (38°13′12″N 116°59′24″E), Hebei Province, China. No endangered or protected species were involved in the field studies. The “List of Protected Animals in China” does not contain the migratory locust *Locusta migratoria* (Orthoptera: Acrididae) which are common insect.

### Insect Rearing

The egg pods of *L. migratoria* were incubated in wet sand in an environmental chamber (Ningbo, China) at 30±1°C with a 14 h:10 h (L: D) photoperiod and 55% humidity. Grasshoppers were transferred to the laboratory after hatching and fed with fresh wheat seedlings, crop leaves and bran [30]. Pots filled with slightly moistened sterile sand were prepared for mature females to deposit their eggs. After oviposition, the egg pods were collected every day and incubated in an environmental chamber as described as above.

### Biotic factors

#### Embryo

The eggs were collected from the fourth day after oviposition until the thirteenth day. Embryo was dissected from eggs in PBS solution (10 mmol Na_2_HPO_4_, 2 mmol/L KH_2_PO_4_, 137 mmol/L NaCl, 2.7 mmol/L KCl, pH 7.4) on ice.

#### Larvae and adult

Samples used in the study comprised 10 first-instar nymphs, 8 second-instar nymphs, 5 third-instar nymphs, 3 fourth-instar nymphs, 2 fifth-instar nymphs, 1 male and female adults (collected at the first day and the tenth day after emergence) for each replication.

#### Tissue

Thirteen tissues and organs were obtained from adults using a dissection needle in ice-cold PBS solution [39]. Tissues included brain, antenna, wings, fore legs, middle legs, hind legs, Malpighian tube, ovary, testis, midgut, epidermis, hemolymph and fat body.

All the samples were kept in −80°C after snap frozen in liquid nitrogen for subsequent RNA extraction. We prepared three biological replications for every sample.

### Abiotic Stresses

#### Insecticide-induced stress

Four insecticides, chlorpyrifos, cyhalothrin, acetamiprid, and chlorantraniliprole, were used in this study. These are commonly used insecticides in Orthoptera pest management programs. The method for insecticide bioassay was the leaf-dip bioassay. Crop discs (3 cm diameter) were dipped for 10 sec in distilled water solutions of formulated insecticide with 0.1% Triton X-100 and air-dried at 25°C for 3 h according to the leaf-dip bioassay [40] which was usually used for insecticide bioassay. The discs were then placed inside transparent plastic cups (6.5 cm×5.0 cm×5.5 cm) covered with clean gauze. For each replication, ten third-instar larvae that had been starved for 6 h were placed in the cup with four discs inside, and three replications were conducted. Five different concentrations were tested for each pesticide. For the controls, the crop discs were dipped in distilled water containing only 0.1% Triton X-100. Other treatments were the same as described above.

The larvae fed on treated crop discs were then reared under normal conditions. After 48 h, we checked for mortality. Mortality data from insecticide bioassays were analyzed for LC_15_ (sublethal dose) values and the SPSS program 17.0 was used to calculate their 95% confidence intervals based on probit analysis (**Table S1**). Third-instar nymphs were subjected to each insecticide with LC_15_ values derived from the toxicity test above. After 48 h rearing under routine conditions, five surviving nymphs were used for RNA extraction as one replication.

#### Temperature-induced stress

Third-instar larvae were transferred directly into thin glass test tubes (2 cm×8 cm) covered by gauze from their rearing conditions (30°C, 14L:12D) for incubation under series of temperatures (0°C, 15°C, 30°C, 36°C, 40°C) for 2 h [41]. For the 0°C incubation, the glass tubes were placed in an ice water mixture. For temperature treatments, five nymphs were used for RNA extraction as one replication.

#### Starvation treatment

Third-instar locust nymphs were placed in glass test tubes without food for 6 h and 12 h. Five nymphs were assembled for RNA extraction as one replication.

### Total RNA Extraction and cDNA Synthesis

The RNeasy Mini Kit (QIAGEN, Germany) was used to extract total RNA from treated samples following the manufacturer's instructions. DNase I (RNase-Free DNase set, QIAGEN, Germany) was used to eliminate DNA contamination according to the recommended procedures. A spectrophotometer (NanoDrop-2000, Thermo Scientific) were used to measure the purity and concentration of total RNA for A260/A280 and A260/A230. The integrity of all RNA samples was then verified immediately via agarose gel electrophoresis. If an 18S band was clearly observed, the RNA samples were considered intact. It could not be identified for the band corresponding to 28S RNA because of the ‘hidden break’ present in insects [42]. M-MLV Reverse Transcriptase (Promega, USA) were used to synthesize complementary DNA (cDNA) from 2 µg of total RNA with Oligo(dT) 18 primer.

### Candidate reference genes selection

Twelve housekeeping genes were selected from previous studies in *L. migratoria* and the LocustDB (http://locustdb.genomics.org.cn/). These genes have been selected as reference genes for normalization factors in *L. migratoria* include *EF1α* (elongation factor 1 alpha), *RPL32* (ribosomal protein L32), *SDH* (succinate dehydrogenase) [37], *His* (histone H3) [43], *Hsp70* (heat shock protein) [44], *Cht2* (probable chitinase 2), *Ach* (acetyl-CoA hydrolase), *18S* (18S rRNA) [38], *RP49* (ribosomal protein 49), *Tub* (*α*-tubulin 1A), *Act* (β-actin), and *GAPDH* (glyceraldehyde-3-phosphate dehydrogenase) [36]. The Primer Premier 5 software (http://www.PremierBiosoft.com/primerdesign/primerdesign.html) was used to design the primers. The parameters in Primer 5 were setting as follows: amplicon length 80–250 bp, melting temperature 58–62°C, primer lengths 15–28 bp, and GC content 40–60% (**Table 1**).

**Table 1:** Details of twelve candidate reference genes used for real-time PCR.

Symbol (AccessionNO.)	Gene name	Description	Primer sequence	Size (bp)	Tm (°C)	E (%)	R^2^
*Ach* (NP-651762)	Acetyl-CoA hydrolase	Involved in acetate utilization in mitochondria	F:CAGTACAAGGAAAACAGCAATAA	126	56.1	102.3	0.999
		and fatty acid metabolism	R: ATCCAGTAAAGGGGCTAAGAA				
*Act* (HQ388822)	β-actin	Cytoskeleton	F:CGAAGCACAGTCAAAGAGAGGTA	156	60.2	94.2	0.999
			R:GCTTCAGTCAAGAGAACAGGATG				
*Hsp70* (AY178988)	Heat shock protein	A member of 70-kDa heat shock protein	F: CTGGTGTGCTCATTCAGGTAT	100	59.7	99.8	0.999
			R: TCGTGGGGCAGGTGGTATT				
*18S* (AF370793)	18SrRNA	Structural constituent of ribosome	F: GCGGTAATTCCAGCTCCAATAG	121	60.1	102.4	0.999
			R: CCCACGATACATGCCAGTTAAA				
*EF1α* (HQ388819)	Elongation factor 1 alpha	Translation elongation factor activity; GTPase	F: AGCCCAGGAGATGGGTAAAG	155	61.9	108.7	0.994
		activity; GTP binding	R: CTCTGTGGCCTGGAGCATC				
*SDH* (HQ388823)	Succinate dehydrogenase	Succinate dehydrogenase activity; Succinate-coA	F:CCACTGAAACTGATCCAAGAGAG	98	60.2	91.5	0.997
		ligase activity; FAD binding;electron carrier activity	R: TCCTGCTCCATTAACTAAGCAAC				
*RPL32* (HQ388820)	Ribosomal protein L32	Structural constituent of ribosome	F: ACTGGAAGTCTTGATGATGCAG	97	60	91.8	0.997
			R: CTGAGCCCGTTCTACAATAGC				
*His* (AF370817)	Histone H3	A protein involved in the structure of chromatin in	F: CACCAAGGCGGCGAGGAAGA	250	64	102	0.997
		eukaryotic cells	R: CGAAGAGGCCGACGAGGTAG				
*Cht2* (NP_477298)	Probable chitinase 2	To hydrolyze chitin	F: CTTCCATTATCCTTGGTTTGC	258	60.1	94.9	0.996
			R: GAGTATGACTGGGTTGTAGCCT				
*GAPDH* (JF_915526)	Glyceraldehyde-3-phosphate	Oxidoreductase in glycolysis & Gluconeogenesis	F: GTCTGATGACAACAGTGCAT	81	61.9	93.2	0.998
	Dehydrogenase		R: GTCCATCACGCCACAACTTTC				
*Tub* (JN676091)	α-tubulin 1A	Cytoskeletal structure protein	F: TGACAATGAGGCCATCTATG	118	55.8	91.3	0.998
			R: CGCAAAGATGCTGTGATTGA				
*RP49* (X00848)	Ribosomal protein 49	Translation	F: CGCTACAAGAAGCTTAAGAGGTCAT	66	61.9	97.3	0.999
			R: CCTACGGCGCACTCTGTTG				
*CHS1* (AF221067.1)	Chitin synthase 1	A key enzymes involved in chitin biosynthesis	F:CTTGAGCCAATTGGTTTGGT	121	57.8	93.1	0.999
			R:TGAGTTCTGTGGATGCAAGG				
*GSTs1* (HM131836)	Glutathione S-transferases	A dimeric enzymes involved in phase II	F:CGCAAGATTAGAATTTGAACAATGGC	181	60.4	96.9	0.998
		detoxification of hydrophobic toxic compounds	R:AGTCTCAGGTCTGTTACAGTCTCAAT				

Size: size of amplicon length; Tm: melt temperature; E: PCR efficiency; R^2^: coefficient of determination.

### Quantitative Real time PCR analysis

An Applied Biosystems 7500 Real-Time PCR System (Applied Biosystems, USA) were used to perform Quantitative Real time PCR (qPCR) experiments in 96-wells reaction plates using SYBR Premix Ex Taq II (Takara, Japan). Each reaction was run in a 20 µL volume reaction [10 µL 2×SYBR Premix Ex Taq II (Tli RNaseH Plus), 0.4 µL ROX Reference Dye II, 6 µL nuclease-free water, 0.8 µL each primer and 2 µL diluted cDNA]. The reaction program was as follows: 95°C 30 s, followed by 40 cycles (95°C for 5 s, 55°C for 30 s and 72°C for 30 s). At the end of each PCR run, a dissociation protocol (melting curve analysis) was applied to all reactions. Each sample was prepared three technical and biological replicates. To estimate amplification efficiency and correlation coefficient (R^2^) of each primer pairs, a range of series dilution of cDNA (10^n^-fold) was used to create the five-point standard curve. The equation (E = [10^(1/-slope)^-1] ×100%) was used to calculate the qPCR efficiency for each primer [45].

### Validation of housekeeping gene selection

To assess the validity of selected internal control genes, the transcription level of the chitin synthase 1 gene (*CHS1*) was estimated for different development stages. We compared the mRNA transcript level of *CHS1* when using only one reference gene [the best (NF_1_) and the worst gene (NF_12_)] and two most stable reference genes (NF_1–2_) recommended by RefFinder. Expression levels of a detoxification-related gene (*GSTs1*) were picked to evaluate the validity of selected reference genes in four tissues (midgut, Malpighian tube, fat body and spermary) and the third-instar larvae subjected to four insecticides. For the tissues, the expression profiles of the gene *GSTs1* were estimated using one reference gene [the most (NF_1_) and the least stable reference gene (NF_12_)] and several stable reference genes (NF_1–2_, NF_1–3_, NF_1–5_) together recommend by RefFinder. For the insecticide treatment, the method to evaluate the expression of *GSTs1* was same as the gene *CHS1* in different development stages. When normalizing using more than one internal reference gene, the geometric mean calculated from the cycle threshold values of the included housekeeping genes was used as the normalization factors (NF_1–n_), and the algorithm (2^−ΔΔCt^) was used to calculate the transcription level of the interested gene. The effect of housekeeping gene selection and usage on the evaluation of interested gene expressions was assessed between those normalized by the least stable reference gene and the recommended combination of reference genes with the highest stability value. T-test was conducted for statistical analysis with software SPSS (ver. 17.0).

### Statistical Analysis

The expression stability of twelve selected internal control genes was evaluated with the delta Ct methods, geNorm v. 3.5 [45], Bestkeeper [46] and NormFinder [47]. At the same time, RefFinder, a comprehensive tool (http://www.leonxie.com/referencegene.php), was adopted to assess and rank the selected housekeeping genes. This tool assigned an appropriate weight to an individual gene and calculated the geometric mean of their weights for the overall final ranking according to the results from each program. Raw Ct values were used for Bestkeeper and RefFinder. For the NormFinder and geNorm software programs, Ct values should be transformed to linear scale expression quantities.

## Results

### The Quality of Total RNA

In this study, the ratio A260/A280 of total RNA obtained from all samples ranged from 1.90 to 2.10 and A260/230 was above 1.90, indicating that all total RNA were adequately free from organic salts and protein contamination. The concentration of total RNA varied from 800 ng/µl to 2000 ng/µl, which was appropriate for synthesizing cDNA template.

### PCR Amplification Efficiencies

For each set of the primer pairs, firstly, twelve candidate reference genes and two target genes were checked by normal PCR which produced a single amplicon with expected size. Then the dissociation curve derived from qPCR with single-peak confirmed the unique amplification and no primer dimer formation. Standard curve method was adopted to calculate the amplification efficiency of each primer pairs with cDNA isolated from third-instar nymphs in ten-fold serial dilution. The PCR efficiency of all the primer pairs ranked from 91.3% (*Tub*) to 108.7% (*EF1α*). The correlation coefficients R^2^ ranged from 0.994 to 0.999 (**Table 1**).

### Expression Profiles of Selected Reference Genes

**Figure 1:**
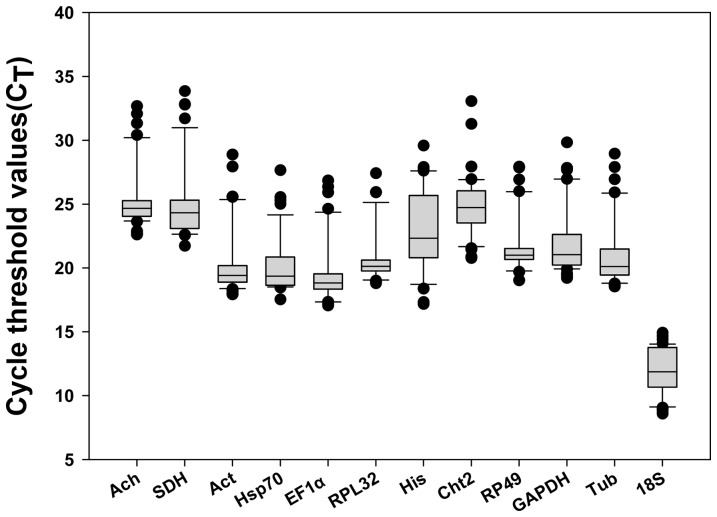
Average cycle threshold (Ct) values of candidate reference genes tested in *Locusta migratoria* under different conditions. The values are the average qPCR Ct values. The dot represents the outliers of replicated samples, while whiskers represent the standard deviation of the mean.

The cycle threshold (Ct) values were adopted to compare the transcript abundance of the selected genes in different samples, assuming equal Ct on behalf of identical transcript amount, since an equal quantity of total RNA were performed in all qPCR reactions. The mean Ct values of the twelve reference genes varied from 11.96 to 25.35, with the lowest and highest Ct values obtained from *18S* (Ct 8.45) and *SDH* (Ct 33.94) (**Figure 1**). *18S* and *EF1α* showed the most abundant expression levels followed by *Hsp70* (mean Ct 20.11), *Act* (mean Ct 20.47), *RPL32* (mean Ct 20.81), *Tub* (mean Ct 21.11), *RP49* (mean Ct 21.72), *GAPDH* (mean Ct 22.05) and *His* (mean Ct 22.97). The moderately abundant transcripts were the two target genes and remaining three reference genes, which had a Ct value of 23 or higher. **Figure 1** also showed that the gene *18S* displayed the lowest dispersion (6.3 cycles) followed by *RPL32* (8.7 cycles). The gene *His* exhibited highest dispersion over all samples indicated by largest whiskers of the box.

**Figure 2:**
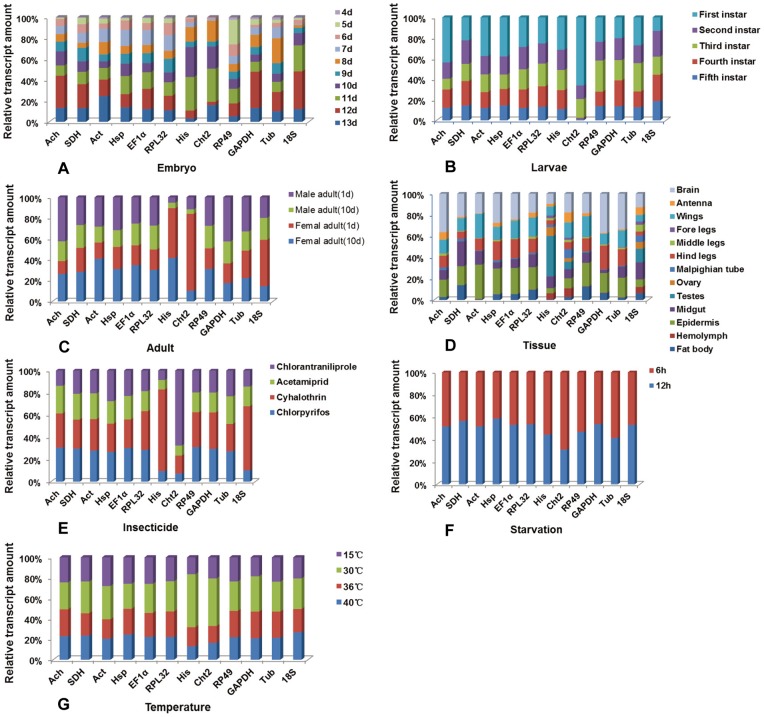
Distribution of relative expression level of the selected control genes across all samples. Expression level is represented as percentages of the aggregated transcript in different experimental conditions pool for each gene. (**A**) embryo; (**B**) nymphs samples; (**C**) adults; (**D**) tissue samples; (**E**) third nymphs subjected to insecticides; (**F**) third nymphs suffering starvation stress; (**G**) third nymphs exposed to temperature treatment.

**Figure 2** revealed the distribution of relative expression level of the selected genes across different samples. For the biotic factors, *RPL32* showed a more constant expression level among samples of different development stages than other candidate genes. The transcript level of *SDH* was also relatively constant in larvae and adult stages. The transcript levels of *His* and *Cht2* were more stable in different tissues than other reference genes. For abiotic factors, the expression level of *Ach* was relatively constant in the third-instar larvae under temperature and starvation stress, while the transcript levels of *SDH* and *Tub* were more constant after insecticide treatment. These results revealed that there was not one reference gene suitable for all biological samples and experimental treatments.

### Expression Stability of Selected Reference Genes

#### Biotic Factors

**Figure 3:**
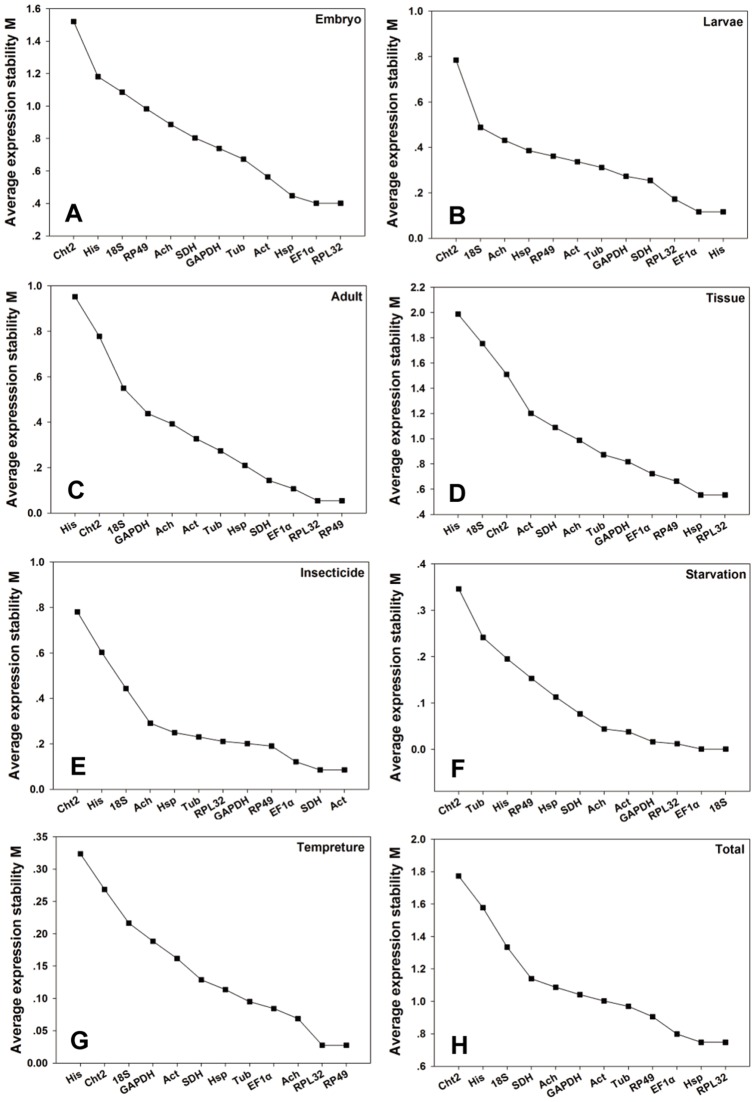
Average expression stability and ranking of twelve candidate reference genes calculated by geNorm. (**A**) embryos samples (**B**) nymphs samples (**C**) adults (**D**) tissue samples (**E**) third instar nymphs subjected to insecticides (**F**) third instar nymphs suffering starvation stress (**G**) third instar nymphs under temperature stress (**H**) all of the biological samples.

**Figure 4:**
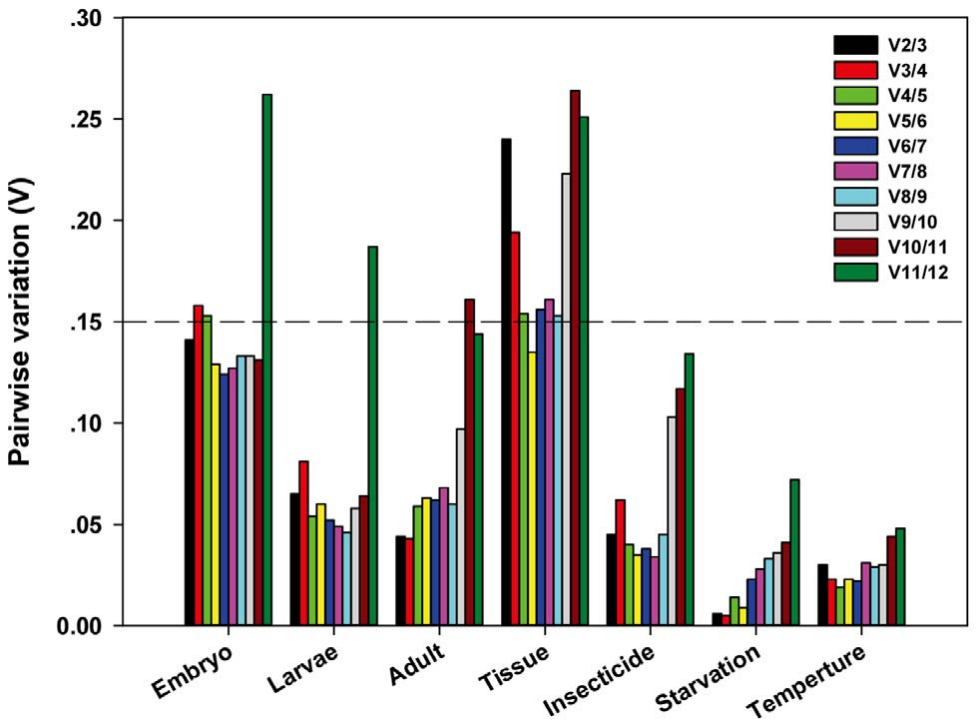
Pairwise variation analysis for an accurate normalization. The pairwise variation analysis is performed by geNorm to determine the optimal number of internal control genes. Each pairwise variation value is compared with 0.15, below which the inclusion of an additional reference gene is not required.

For embryo study, the overall ranking order of the best-suited reference genes generated by all the programs, except for Bestkeeper, were coherent, though the stability rankings fluctuated among separated analyses to some extent. Three methods (geNorm, NormFinder, and delta Ct methods) identified the top four ranked genes as *RPL32*, *Hsp70*, *EF1α* and *Act* for embryo, while Bestkeeper allocated *RP49*, *RPL32*, *Tub* and *GAPDH* as the four best-suited genes (**Figure 3**, **Table 2**). Interestingly, *RPL32* was predicated stable by all software packages and *EF1α* by three programs. Our results displayed *Cht2* as the least stable gene for embryo. RefFinder analysis showed the most stable genes were ranked as follows: *Cht2* < *His* < *18S* < *Ach* < *GAPDH* < *SDH* < *Rp49* < *Tub* < *Act* < *Hsp70* < *RPL32* < *EF1α* (**Table 2**). According to the analysis of the pairwise variation, the V_2/3_-value was below the default value (0.15) (**Figure 4**). This indicated that the addition of a third gene didn't have great effect on normalization process. Therefore, two reference genes were appropriate to normalize gene expression.

**Table 2:** Expression stability of the candidate reference genes under biotic conditions.

Biotic	Reference	Delta Ct		Bestkeeper			Genorm		Normfinder		RefFinder	
Condition	Gene	StdDev	Rank	SD	R	Rank	M	Rank	SV	Rank	Stability	Rank
Embryo	*18S*	1.606	9	1.550	0.831	10	1.086	10	0.851	10	9.740	10
	*Ach*	1.335	8	1.449	0.969*	9	0.887	8	0.493	5	7.326	9
	*Act*	1.188	4	0.886	0.854	6	0.563	4	0.486	4	4.899	4
	*Cht2*	3.112	12	3.084	0.938*	12	1.521	12	2.168	12	12.000	12
	*EF1α*	1.048	1	0.848	0.961*	5	0.401	1	0.226	2	1.778	1
	*GAPDH*	1.327	7	0.841	0.794	4	0.739	6	0.649	8	6.055	8
	*His*	1.632	11	1.552	0.829	11	1.181	11	0.812	9	10.462	11
	*Hsp70*	1.103	2	1.000	0.932*	7	0.447	3	0.025	1	2.546	3
	*RP49*	1.625	10	0.652	0.508	1	0.983	9	0.991	11	5.609	6
	*RPL32*	1.118	3	0.777	0.911*	2	0.401	1	0.399	3	2.060	2
	*SDH*	1.245	5	1.054	0.927*	8	0.803	7	0.506	6	5.789	7
	*Tub*	1.297	6	0.834	0.814	3	0.673	5	0.574	7	5.010	5
Larvae	*18S*	0.867	11	0.307	−0.458	4	0.488	11	0.587	11	8.542	10
	*Ach*	0.748	10	0.561	0.800*	11	0.431	10	0.386	10	10.241	11
	*Act*	0.554	4	0.413	0.956*	9	0.337	7	0.069	3	5.244	6
	*Cht2*	2.097	12	2.044	0.842*	12	0.784	12	1.554	12	12.000	12
	*EF1α*	0.497	1	0.316	0.980*	5	0.116	1	0.040	1	2.115	1
	*GAPDH*	0.608	8	0.222	0.502	1	0.272	5	0.316	9	2.913	3
	*His*	0.519	3	0.362	0.968*	6	0.116	1	0.041	2	3.663	5
	*Hsp70*	0.606	7	0.414	0.847*	10	0.385	9	0.198	6	7.841	9
	*RP49*	0.641	9	0.387	0.549	7	0.362	8	0.243	7	7.707	8
	*RPL32*	0.508	2	0.231	0.890*	2	0.172	3	0.082	4	2.632	2
	*SDH*	0.588	6	0.270	0.647	3	0.254	4	0.283	8	3.464	4
	*Tub*	0.573	5	0.409	0.755*	8	0.312	6	0.131	5	5.886	7
Adult	*18S*	0.983	10	0.481	0.215	8	0.550	10	0.524	9	9.212	10
	*Ach*	0.863	9	0.567	0.056	9	0.392	8	0.560	10	8.972	9
	*Act*	0.767	7	0.575	0.504	10	0.327	7	0.390	7	7.653	7
	*Cht2*	1.597	11	1.201	0.735	11	0.777	11	1.140	11	11.000	11
	*EF1α*	0.634	5	0.286	0.360	5	0.107	3	0.229	6	4.606	6
	*GAPDH*	0.817	8	0.430	−0.012	7	0.438	9	0.468	8	7.969	8
	*His*	1.651	12	1.547	0.726	12	0.952	12	1.186	12	12.000	12
	*Hsp70*	0.631	4	0.384	0.722	6	0.210	5	0.114	3	4.356	5
	*RP49*	0.579	1	0.241	0.660	3	0.054	1	0.195	4	1.861	2
	*RPL32*	0.613	3	0.222	0.178	2	0.054	1	0.221	5	2.340	3
	*SDH*	0.600	2	0.147	0.598	1	0.143	4	0.058	1	1.682	1
	*Tub*	0.652	6	0.267	0.551	4	0.273	6	0.109	2	4.120	4
Tissue	*18S*	2.634	11	0.466	0.323	1	1.754	11	1.665	11	6.040	6
	*Ach*	1.885	7	2.757	0.929*	8	0.988	7	1.011	7	7.238	9
	*Act*	2.020	9	3.098	0.954*	12	1.201	9	1.080	9	9.671	12
	*Cht*	2.389	10	0.836	0.645	2	1.510	10	1.403	10	6.687	8
	*EF1α*	1.547	4	2.854	0.992*	10	0.722	4	0.629	4	5.030	4
	*GAPDH*	1.697	6	2.777	0.959*	9	0.818	5	0.814	6	6.344	7
	*His*	3.078	12	1.124	0.014	3	1.987	12	2.063	12	8.485	10
	*Hsp70*	1.497	2	2.405	0.974*	4	0.554	1	0.494	3	2.213	2
	*RP49*	1.504	3	2.482	0.982*	6	0.663	3	0.433	2	3.224	3
	*RPL32*	1.462	1	2.440	0.976*	5	0.554	1	0.429	1	1.495	1
	*SDH*	1.922	8	3.003	0.950*	11	1.089	8	1.050	8	8.663	11
	*Tub*	1.615	5	2.552	0.958*	7	0.873	6	0.656	5	5.692	5

StdDev, standard deviation; SD, standard deviation; SV, stability value; r, Pearson correlation coefficient; *p≤0.001.

For Larvae analysis, the rankings of the best-suited reference genes obtained by the delta Ct approach and geNorm were similar. Additionally, the top two ranked genes identified by geNorm were the same as those generated by NormFinder. The result from Bestkeeper was different from those generated by four other methods. Namely, the delta Ct method and other two programs ranked *EF1α* as the most stable gene, while *GAPDH* was ranked in the top position according to Bestkeeper (**Table 2**). *Cht2* appeared as the least stable gene for Larvae. RefFinder analysis showed the most stable genes were ranked as follows: *Cht2* < *Ach* < *18S* < *Hsp70* < *RP49* < *Tub* < *Act* < *His* < *SDH* < *GAPDH* < *RPL32* < *EF1α* (**Table 2**). According to the analysis of the pairwise variation, the V value (V_2/3_) was less than 0.15 (**Figure 4**). This indicated that the addition of a third gene didn't have a great effect on normalization process. Therefore, two reference genes were appropriate to normalize gene expression.

For adult assessment, it was similar for the rankings of the best-suited reference genes got by the Delta Ct approach and Bestkeeper. They placed *RP49*, *SDH*, and *RPL32* on the top three positions, although the rank order was slightly altered (**Table 2**). The top three genes identified by geNorm and NormFinder were largely different from the results generated by delta Ct method. Additionally, the most stable genes from geNorm also differed from those selected by NormFinder. NormFinder placed the gene *SDH* on the top position followed by *Tub* and *Hsp70*, while geNorm select *RPL32* and *RP49* as the most appropriate candidate genes with the lowest M value (0.054) (**Table 2** **,** **Figure 3**) and *EF1α* was placed on the third position with the M value (0.107). At the same time, *His* appeared as the least stable gene by four methods for adult. RefFinder analysis showed the most stable genes were ranked as follows: *His* < *Cht2* < *18S* < *Ach* < *GAPDH* < *Act* < *EF1α* < *Hsp70* < *Tub* < *RPL32* < *RP49* < *SDH* (**Table 2**). According to the analysis of the pairwise variation, the V value (V_2/3_) was below 0.15 (**Figure 4**). This indicates the best normalization factors for gene transcript analysis should contain at least two reference genes.

For tissue assay, the top ranked four genes exhibited by delta Ct method for different tissues were *RPL32*, *Hsp70*, *RP49* and *EF1α*, which is similar to the results generated by NormFinder and geNorm (**Table 2**). Additionally, *RPL32* was considered as the most appropriate gene by these three algorithms. However, Bestkeeper analysis identified *18S* as the best one, followed by *Cht2* and *His*. The gene *Hsp70* was ranked as fourth. Likewise, the delta Ct method, geNorm and Normfinder ranked *His* as the least stable gene, but Bestkeeper selected *Act* as the least stable gene for all tissue samples. RefFinder analysis ranked the best suitable candidate reference genes as follows: *Act* < *SDH* < *His* < *Ach* < *Cht2* < *GAPDH* < *18S* < *Tub* < *EF1α* < *Rp49* < *Hsp70* < *RPL32* (**Table 2**). According to the analysis of the pairwise variation, the value of V_2/3_, V_3/4_ and V_4/5_ were all above the proposed value (0.15), but the V_5/6_ value was under the threshold value of 0.15. This indicates the best normalization factors for gene transcript analysis should contain at least five reference genes.

#### Abiotic stresses

For assay pretreated with different insecticide, it was similar for the top ranked three genes exhibited by the delta Ct approach and NormFinder, though the rank order was slightly altered. The delta Ct methods selected *Act* as the most suitable normalization factor for qPCR normalization followed by *RPL32* and *RP49* (**Table 3**). *RPL32* was identified as the best endogenous control gene by NormFinder, while *Act* and *RP49* were ranked in the second and third position, respectively. However, geNorm and Bestkeeper analysis generated different results. The top three genes identified by geNorm were *SDH*, *Act* and *EF1α* (**Figure 3**), but *Hsp70* was selected as the most stable gene by Bestkeeper analysis followed by *Tub* and *Act*. RefFinder analysis showed the most stable genes were ranked as follows: *Cht2* < *His* < *18S* < *Ach* < *GAPDH* < *EF1α* < *Tub* < *RP49* < *RPL32* < *Hsp70* < *SDH* < *Act* (**Table 3**). According to the analysis of the pairwise variation, the V value (V_2/3_) was less than 0.15 (**Figure 4**). Thus, two reference genes were sufficient to normalize gene expression.

**Table 3:** Expression stability of the candidate reference genes under abiotic conditions.

Abiotic	Reference	Delta Ct		Bestkeeper			geNorm		Normfinder		RefFinder	
Condition	Gene	StdDev	Rank	SD	R	rank	M	Rank	SV	Rank	Stability	Rank
Insecticide	*18S*	0.995	10	0.776	0.900*	10	0.443	10	0.554	10	10.000	10
	*Ach*	0.679	9	0.394	0.446	8	0.291	9	0.321	9	8.739	9
	*Act*	0.536	1	0.188	0.693	3	0.0852	1	0.120	2	1.565	1
	*Cht2*	1.551	12	1.116	0.043	11	0.781	12	1.023	12	11.742	12
	*EF1α*	0.603	5	0.249	0.511	5	0.121	3	0.226	7	5.439	7
	*GAPDH*	0.626	8	0.381	0.784	7	0.201	5	0.214	6	7.200	8
	*His*	1.296	11	1.146	0.843*	12	0.602	11	0.833	11	11.242	11
	*Hsp70*	0.605	6	0.155	0.512	1	0.249	8	0.181	4	3.130	3
	*RP49*	0.574	3	0.371	0.835*	6	0.190	4	0.153	3	4.243	5
	*RPL32*	0.567	2	0.395	0.911*	9	0.211	6	0.110	1	3.350	4
	*SDH*	0.581	4	0.201	0.432	4	0.0852	1	0.211	5	2.991	2
	*Tub*	0.609	7	0.172	0.074	2	0.230	7	0.233	8	4.281	6
Starvation	*18S*	0.329	2	0.089	−0.197	3	0.001	1	0.085	3	2.711	3
	*Ach*	0.333	3	0.051	−0.437	1	0.044	6	0.078	2	1.565	2
	*Act*	0.328	1	0.053	−0.209	2	0.038	5	0.061	1	1.189	1
	*Cht2*	0.751	12	0.576	−0.045	12	0.346	12	0.490	12	12.000	12
	*EF1α*	0.368	4	0.156	0.668	4	0.001	1	0.119	4	4.000	4
	*GAPDH*	0.523	11	0.374	0.614	11	0.017	4	0.295	11	10.462	11
	*His*	0.502	9	0.269	0.287	9	0.195	10	0.264	9	9.240	9
	*Hsp70*	0.422	5	0.254	0.114	8	0.113	8	0.221	7	6.117	5
	*RP49*	0.456	8	0.207	0.268	5	0.153	9	0.216	6	6.620	7
	*RPL32*	0.435	6	0.231	0.741	7	0.012	3	0.211	5	6.192	6
	*SDH*	0.443	7	0.216	0.382	6	0.077	7	0.233	8	6.701	8
	*Tub*	0.511	10	0.345	0.655	10	0.242	11	0.274	10	10.241	10
Temperature	*18S*	0.374	10	0.241	0.565	8	0.217	10	0.211	10	9.457	10
	*Ach*	0.261	6	0.086	0.371	2	0.069	3	0.139	7	3.984	6
	*Act*	0.308	9	0.256	0.743	10	0.161	8	0.145	8	8.712	9
	*Cht2*	0.458	11	0.444	0.956 *	11	0.268	11	0.294	11	11.00	11
	*EF1α*	0.241	4	0.018	0.716	3	0.084	4	0.088	5	3.936	4
	*GAPDH*	0.299	8	0.250	0.845 *	9	0.188	9	0.126	6	7.896	8
	*His*	0.561	12	0.513	0.967	12	0.324	12	0.398	12	12.00	12
	*Hsp70*	0.298	7	0.049	−0.326*	1	0.114	6	0.181	9	4.409	7
	*RP49*	0.238	3	0.128	0.713	5	0.028	1	0.084	4	2.783	2
	*RPL32*	0.235	1	0.127	0.755*	4	0.028	1	0.068	3	1.861	1
	*SDH*	0.241	5	0.140	0.917*	7	0.129	7	0.038	1	3.956	5
	*Tub*	0.235	2	0.129	0.853 *	6	0.095	5	0.065	2	3.310	3

StdDev, standard deviation; SD, standard deviation; SV, stability value; r, Pearson correlation coefficient; *p≤0.001.

For survey after starvation, the top ranked four genes exhibited by Normfinder were *Act*, *Ach*, *18S* and *EF1α*, which was the same as the results generated by Bestkeeper (**Table 3**). What's more, the ranking orders of these four stable genes were also the same. Additionally, the best-suited reference genes generated by delta Ct analysis were also these four genes, though the stability rankings were slightly different. GeNorm analysis selected *18S* and *EF1α* as the best suitable reference genes (**Figure 3**). The gene *RPL32* was ranked in the third position followed by *GAPDH*. Interestingly, the candidate gene *Cht2* was considered as the most unstable gene by all the algorithms. RefFinder analysis showed the most stable genes were ranked as follows: *Cht2* < *GAPDH* < *Tub* < *His* < *SDH* < *RP49* < *RPL32* < *Hsp70* < *EF1α* < *18S* < *Ach* < *Act* (**Table 3**). The V value (V_2/3_) was under the proposed value (0.15) (**Figure 4**). This indicated that the addition of a third gene didn't have a great effect on normalization process. Therefore, two reference genes were appropriate to normalize gene expression.

For analysis of variant temperature, the most stable reference gene selected by the delta Ct methods was *RPL32*, which also the best gene identified by geNorm (**Table 3**). The delta Ct methods identified *Tub* as the second most stable gene followed by *RP49*, while geNorm identified *RP49* and *RPL32* both as the best suitable genes followed by *Ach* (**Figure 3**). According to the results of Normfinder analysis, the gene *RPL32* was ranked in the third position. The gene *SDH* appeared as the most stable gene followed by *Tub*. The Bestkeeper analysis generated different results compared to the three other algorithms. Bestkeeper identified *Hsp70* as the most suitable gene for normalizing gene expression and *Ach* was set in the second position followed by *EF1α*. All the statistical algorithms indicated *Cht2* and *His* as the two least stable genes. RefFinder analysis showed the expression stability of genes was ranked as follows: *His* < *Cht2* < *18S* < *Act* < *GAPDH* < *Hsp70* < *Ach* < *SDH* < *EF1α* < *Tub* < *RP49* < *RPL32* (**Table 3**). According to the analysis of the pairwise variation, the V_2/3_-value was under the threshold value (0.15) (**Figure 4**). Therefore, two reference genes were appropriate to normalize gene expression.

For the total investigated samples, the rankings of candidate reference gene stability obtained by three algorithms containing the delta Ct approach, Normfinder and geNorm were largely similar. These three methods indicated the same three most stable candidate genes (*EF1α*, *Hsp70*, *RPL32*), though the ranking order of these three reference genes were different. However, Bestkeeper generated different results: *RPL32* and *RP49* appeared as the two best normalization factors followed by *18S*. Likewise, all the programs, except for Bestkeeper, selected *Cht2* as the worst gene. *His* was selected as the most unstable reference gene by Bestkeeper. RefFinder analysis showed the most stable genes were ranked as follows: *His* < *Cht2* < *SDH* < *GAPDH* < *18S* < *Act* < *Ach* < *Tub* < *RP49* < *EF1α* < *RPL32* < *Hsp70* (**Table S2**).

### Validation of reference gene selection

**Figure 5:**
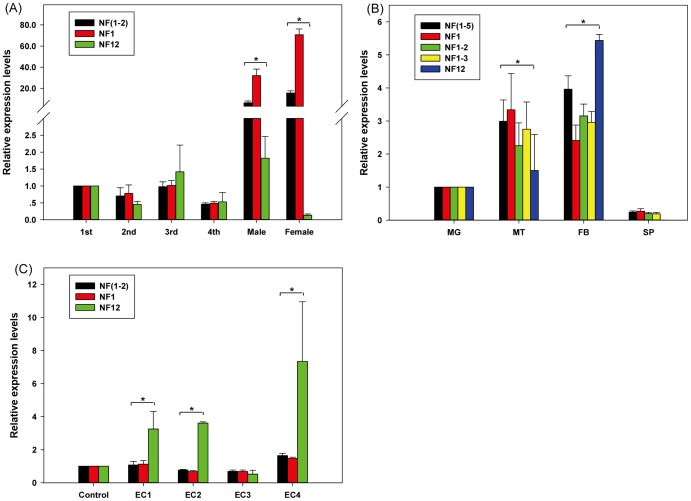
Validation of reference gene selection. (**A**) Relative Expression levels of *CHS1* in six developmental stages (**B**) Relative Expression levels of target gene, *GSTs1*, in midgut (MG), Malpighian tubules (MT), fatbodies (FB), Spermary (SP) (**C**) and different insecticides, cyhalothrin (EC1), acetamiprid (EC2), chlorantraniliprole (EC3), chlorpyrifos (EC4). NF1, using the single one best reference gene for normalization; NF (1–2), using two best reference genes; NF (1–3), using three best reference genes; NF (1–5) using four best reference genes; NF (12), using the least stable reference gene. Asterisks indicate significant difference of the expression of the target gene based on three biological replications. (*P*<0.05, t-test; n = 3).

To assess the performance of selected reference genes, the transcript level of two target genes (*CHS1*, *GSTs1*), were assessed under various experimental conditions. For different development stages, using the best reference gene or the recommended two most stable references to normalize, *CHS1* transcript level were higher in adult compared to larvae stages (**Figure 5A**). However, when the most unstable gene was used to normalize, no evident difference was detected. We also found that the expression level of *CHS1* normalized against the combination of two best reference genes was very different from the least stable reference gene (*P*<0.05) (**Figure 5A**). For qPCR data analysis in different tissues, if normalized using more than one reference gene (*RPL32*+*Hsp*, *RPL32*+*Hsp*+*RP49*, *RPL32*+*Hsp*+*RP49*+*EF1α*+*Tub*), the expression profile of *GSTs1* in Malpighian tube and fat body was similar (**Figure 5B**) and higher compared to the other tissues. Using the single best reference gene as a normalization factor, similar results were found in the Malpighian tube and fat body. However, the transcript level of *GSTs1* was decreased in Malpighian tube when normalized against the least stable reference gene (*His*) (*P*<0.05) (**Figure 5B**). For insecticide treatment, when normalized against the combination of recommended reference genes (*RPL32*, *Act*), the expression of *GSTs1* was increased by 1.07-fold for treatment of Cyhalothrin and 1.64 fold for Chlorpyrifos compared with the control insects, but was reduced for the other insecticides. Using the single best reference gene (*RPL32*) for normalization, imilar expression levels were observed. However, notable differences were found when normalized against the least stable gene (*Cht2*) (**Figure 5C**).

## Discussion

As an accurate and sensitive method to quantify mRNA transcription levels, qPCR has played a very important role in molecular biology research. Normalization with endogenous reference genes is one very important factor which affects the correct measurement of gene transcript level changes. Researchers are required to prove that the candidate reference gene of choice is appropriate for data normalization of differentially expressed genes under a specific experimental condition. In this report, twelve candidate reference genes in *L. migratoria* were assessed for expression stability under the context of different development stages, tissues and special experiment treatments (insecticide, temperature and starvation).

The efficiencies of amplification of reference genes are directly related to the quality of results obtained from qPCR. Therefore, we calculated the efficiencies of each candidate gene prior to quantification. Twelve candidate genes were screened out with amplification efficiency above 91%, while genes with extreme efficiency value were excluded for further analysis.

The expression stability of selected internal control genes was evaluated with four statistical algorithms (the delta Ct approach, geNorm, Bestkeeper, and NormFinder). We assumed that a combination of different mathematical models enabled a better evaluation of the most reliable reference genes. However, there were discrepancies of best reference genes and the stability rankings among the different programs (**Table 2** **,** **Table 3**). This was caused by different mathematical models adopted by each program [47]–[49]. To solve this problem, RefFinder was adopted to comprehensively evaluate and rank reference genes. To evaluate reliable reference genes using different strategies, we need to be aware of their property. GeNorm software is one commonly used statistical method. The underlying principle of this method is that the transcript ratio of two best reference genes should be same in all test samples. This program ranks the reference gene via calculating M value (the average expression stability) for each gene. At the same time, it also revealed the optimal number of reference gene by analyzing the pairwise variation (V). A V score of less than 0.15 was ideal for valid normalization [46]. However, the disadvantage of geNorm was its sensitivity to co-regulation. It usually select genes with the highest degree of similarity in their transcript level [47].

Compared to geNorm, NormFinder was lack of sensitivity to co-regulation of the reference gene. When using the gene to normalize, NormFinder could supply an expression stability value for each gene that empowers the user to analyze the systematic error.

BestKeeper is another Excel-based tool that evaluates expression stability of reference genes based on two variations (SD and CV values). Genes with an SD value higher than 1 were unacceptable. It then determines the best reference gene according to the probability (*p*) value and the *Pearson correlation coefficient (r)* calculated from *pair-wise correlation analysis* of all pairs of selected reference genes [45]. Our results revealed that the rank of most suitable genes obtained from BestKeeper was slightly different from other statistical algorithms. As shown in Table 2, *EF1α* was ranked at the top position by three programs for embryo stage and ranked fifth by BestKeeper. A similar situation was observed with larvae and tissue samples. This might be caused by the statistical algorithms used by BestKeeper that combines highly correlated reference genes into an index. It usually represents the average of the best suitable reference genes. Therefore, BestKeeper may not be able to sensitively distinguish between stable and unstable reference genes [45], [50], [51].

The delta Ct approach is similar to that described by the report of Vandesompele for geNorm program, whereby ‘pairs of genes’ are compared [50]. This method uses delta Ct comparisons between genes to bypass the need to accurately quantify input RNA.

Consistent with the reports in *Drosophila* [51] and *Spodoptera litura* [52], this study revealed that it's difficult to identify a universally appropriate reference genes for qPCR analyses, as all the selected reference genes exhibited notable variation of transcript levels under different experimental conditions. Furthermore, the best recommended reference genes were also different. According to RefFinder, which gave the overall final ranking based on the results from each program, *EF1α* was considered as the best reference gene in embryo and nymph stages, *SDH* was selected as the most appropriate gene in adult stage, *Act* was selected as the best gene in insecticide and starvation treatments, and *RPL32* appeared to be the best normalization factor in different tissues and temperature treatments.

*EF1α*, one kind of protein that contributed to binding aminoacyl-transfer RNA to ribosomes during protein synthesis [53], was placed on the top position for the stability ranking in embryo and larvae stage and ranked as the forth stable gene in tissues samples, starvation and temperature treatment. These results showed very similar correlations with the research in Drosophila across abiotic stress. *EF1α* was assumed to be the best gene in the brain of fifth-instar nymph of *Schistocerca gregaria* and *Chortoicetes terminifera* reared under different density treatments. Surprisingly, *EF1α* was not a good choice for adult according to the analysis of all programs except geNorm. This also highlighted the necessity for validation of the reference genes for different development stages.

*Act* plays a key role in cytoskeleton maintenance and cell motility. It is the most abundant protein in eukaryotic cells [54]. Although *Act* has been usually used as a normalization factor in molecular expression studies [55], several studies have shown that the expression of *Act* fluctuated with aging, growth [56], [57], developmental stage and differentiation [58], [59]. Our study also found *Act* displayed very low stability in different development stages and tissues. However, *Act* was still found to be the most reliable marker of internal control in the insecticide and starvation treatment.

*RPL32* (ribosomal protein L32) is a ribosomal structural constituent. In this study, *RPL32* was selected as the most appropriate reference gene in different tissues and temperature treatment. *RPL32* also appeared as the second most suitable reference gene in embryo and nymphs stage. Our conclusions were in accordance with the research in *Chortoicetes terminifera* [37] and corpora allata of *Diploptera punctate* [60]. Additionally, the stability of *RP49* was always behind *RPL32* in our study, though they are both ribosomal proteins. *Tub*, a type of cytoskeletal structure protein, is another commonly used reference gene. In this study, *Tub* was identified as a moderately stable gene with stability rank around fifth in most samples except for temperature and starvation treatments. *Tub* appeared as the third most appropriate reference gene in third-instar nymphs subjected to temperature treatment and a variable gene under starvation stress. To the best of our knowledge, *Tub* has been reported unsuitable to normalize gene expression in the brain of desert locust [36] and in virus-infected planthoppers [61].

*SDH* and *GAPDH* are two multifunctional enzymes involved in citrate cycle and metabolic pathways, respectively. Our results showed *SDH* was the best reference gene for adult stage and insecticide treatment. We also found that *SDH* and *GAPDH* were stable in larvae stage followed by *EF1α* and *RPL32*. For other experimental conditions, *SDH* and *GAPDH* were not good choices, especially for the abiotic stress. To our knowledge, the expression level of genes which participated in metabolic processes might fluctuate largely under heat stress [62], and *GAPDH* should be avoided to normalize gene expression in hypoxia experiments [63]. *Hsp70*, involved in translating one kind of 70 kDa heat shock protein [64], was chosen as the reference gene to assess the expression of *AChE* gene (acetylcholinesterase) after injection with dsRNA in *L. migratoria manilensis* [44]. In this study, *Hsp70* was identified as the second most appropriate candidate gene in different tissues and third most stable in embryo stage and larvae treated with different insecticides. However, *Hsp70* was detected to be unstable in larvae treated at different temperatures as it is sensitive to temperature. *His*, the housekeeping gene histone H3 which encodes histone protein [65], was rarely used as a normalization gene in insect. According to our study, *His* appeared as the second most suitable gene based on the assessment of NormFinder and geNorm in nymph stage. However, it was the most variable gene in other conditions.

The *18S* ribosomal subunit was highly expressed in all samples with the lowest Ct values. The low Ct values reflect the large quantity of transcripts. mRNA only constitutes 5% of the total RNA, while rRNA corresponds to a large portion of the RNA. Therefore, it might not a good idea to choose *18S rRNA* as the internal control factor. Interestingly, our study indicated that *18S* was not stable enough based on the analysis of all the programs except for Bestkeeper. Many previous studies also showed that *18S* was not a suitable reference gene [66]–[71]. Therefore, we did not recommend *18S rRNA* to normalize gene expression in our experimental conditions.

*Cht2* and *Ach* were used as two novel reference genes identified from the locust microarray data [38]. *Ach* is involved in fatty acid metabolism [72] and acetate utilization in mitochondria [73]. In this study, *Ach* was selected as the best gene in third-instar nymphs suffering starvation. However *Cht2*, which plays a role in hydrolyzing chitin, was considered as the worst gene in all samples in this study. The stability of *Cht2* had also been found unreliable for gene expression analysis in a previous study in locusts exposed to hypobaric hypoxia [38].

To accurately measure the expression levels of a target gene, normalization by multiple housekeeping genes is necessary. However, it is impractical to quantify more stable reference genes than necessary, especially when the amounts of template are limited. Vandesompele et al. [46] recommended to determine the ideal number of selected housekeeping genes by calculating the normalization factor (NF) with geNorm. If the pairwise variation (V_n/n+1_) was below 0.15, it means adding n+1 gene has no obviously effect on normalization factors. Then, the geometric average of the top n candidate reference genes in the system would be the optimal normalization factor for the future experiment [46]. In our study, the V_2/3_ values were all below 0.15 for the development stages and abiotic stress, so two best reference genes are sufficient to analysis the expression of the gene of interest. **Figure 5** showed that two most appropriate genes provided a more conservative estimation of target gene transcription compared to a single gene. Our results also demonstrated that the application of the least stable reference gene could result in false interpretation (**Figure 5A**). As for the different tissues, V_5/6_ was below the proposed 0.15 value, so the best number of selected reference genes should be five. However, it will require a large amount of resources using five control genes as a normalization factor, and our results demonstrated that the expression level and pattern of target gene *GSTs1* in tissues were similar when normalized against three best reference genes and five most stable reference genes (**Figure 5B**). Therefore, we believe that using three best control genes is a valid normalization strategy for tissue samples.

As a proof of principle, our validation results were tested by evaluating the transcript of two target genes in different development stages, tissues and larvae subjected to insecticide treatment. *CHS1* plays an important role in chitin synthesis in insect cuticle [74]. The gene *CHS1* of *L. migratoria* is expressed consistently in every life stage and with the highest transcript amount in adults [75]–[77]. In this report, the expression level of *LmCHS1* was highest in adult when normalized using the two best reference genes (**Figure 5A**), but not when using the least stable reference gene for normalization. Glutathione *S*-transferases (GSTs), a diverse family of dimeric enzymes, can eliminate toxicants from a cell [78]–[79]. Our results demonstrated that the highest transcript levels of *GSTs1* mRNA were detected in fat body and Malpighian tubules when using the recommended set of reference genes for normalization (**Figure 5B**). This can be explained by the fact that the fat body of insects is the main metabolic detoxification center [76]. Our results were also in accordance with a previous report [77]. However, when only normalized by the least stable reference gene, the expression pattern of *GSHs1* was very different. Similar results were observed when evaluating the expression level of *GSHs1* in the third-instar larvae subjected to insecticides (**Figure 5C**). Therefore, using appropriate reference genes for normalization would be one of the key factors for accurate estimation of target gene expression, while unsuitable normalization factors might lead to deviated results and concealing of true outcome.

## Conclusions

In conclusion, our study provides a comprehensive assessment for the suitable reference genes for qPCR in *L. migratoria* across all the development stages, tissues and three abiotic stress: *EF1α* and *RPL32* were found to be reliable for embryo and larvae stage; *SDH* and *RP49* were optimal for adult stage; *RPL32*, *Hsp70* and *RP49* should be recommended for study in different tissues; *Act* and *SDH* would be appropriate for larvae treated with insecticide treatment; *Act* and *Ach* should be used for larvae suffering starvation; and *RPL32* and *RP49* were selectable for larvae subjected to different temperature treatment (**Table 4**). Our data verified the caution that the expression stability of selected reference genes needed to be evaluated in different treated samples. This study will benefit future work on target gene expression in *L. migratoria*.

**Table 4:** Preferable control genes in *L. migratoria* across different experimental conditions.

Experimental conditions		Preferable reference genes		
Biotic factors	Embryo	*EF1α*	*RPL32*	
	Larvae	*EF1α*	*RPL32*	
	Adult	*SDH*	*RP49*	
	Tissue	*RPL32*	*Hsp70*	*RP49*
Abiotic factors	Insecticide	*Act*	*SDH*	
	Starvation	*Act*	*Ach*	
	Temperature	*RPL32*	*RP49*	

## Supporting Information

Table S1 The insecticide bioassay to the third-instar nymphs of *Locusta migratoria*.
(DOC)

Table S2 Expression stability of the candidate reference genes across all the samples.
(DOCX)
