# Supplementary material for: Selection and Assessment of Reference Genes for Quantitative PCR Normalization in Migratory Locust Locusta migratoria (Orthoptera: Acrididae)
Source: PLoS One. 2014 Jun 2;9(6):e98164. doi: 10.1371/journal.pone.0098164 (PMC4041718; doi:10.1371/journal.pone.0098164)
Supplement: Table S1 — The insecticide bioassay to the third-instar nymphs of Locusta migratoria. (DOC) [file pone.0098164.s001.doc]

**Table S1.** The toxicity of insecticides to the third-instar larvae of *L. migratoria*.

| **Insecticides** | **N^a^** | **Slope±SE^b^** | **LC15^c^** | **LC50^c^** | **Χ^2d^** |
| --- | --- | --- | --- | --- | --- |
| Chlorpyrifos | 180 | 3.655±0.543 | 2.707（1.831, 3.439） | 5.200（4.190, 6.595） | 10.653 |
| Cyhalothrin | 180 | 1.958±0.298 | 0.473（0.260, 0.682） | 1.602（1.215, 2.119） | 6.132 |
| Acetamiprid | 180 | 2.695±0.533 | 0.264（0.145, 0.353） | 0.640（0.527, 0.782） | 12.936 |
| Chlorantraniliprole | 180 | 2.201±0.404 | 1.379（0.643, 2.051） | 4.078（3.000, 5.303） | 4.122 |

^a^Number of tested larvae; ^b^SE=standard error; ^c^Expressed in mg/l; 95% fiducial limits (FL) of LC_15_, LC_50_ are given in parenthesis, respectively. ^d^Chi-square testing linearity of dose-mortality responses
