# Supplementary material for: Selection and Assessment of Reference Genes for Quantitative PCR Normalization in Migratory Locust Locusta migratoria (Orthoptera: Acrididae)
Source: PLoS One. 2014 Jun 2;9(6):e98164. doi: 10.1371/journal.pone.0098164 (PMC4041718; doi:10.1371/journal.pone.0098164)
Supplement: Table S2 — Expression stability of the candidate reference genes across all the samples. (DOCX) [file pone.0098164.s002.docx]

**Table S2.** Expression stability of the candidate reference genes across all the samples.

| **Total** | **Reference** | **Delta Ct** | | **Bestkeeper** | | | **geNorm** | | | **Normfinder** | | **RefFinder** | |
| --- | --- | --- | --- | --- | --- | --- | --- | --- | --- | --- | --- | --- | --- |
|  | **Gene** | **StdDev** | **Rank** | **SD** | **R** | **rank** | **M** | **Rank** | | **SV** | **Rank** | **Stability** | **Rank** |
|  | *18S* | 2.136 | 10 | 1.591 | 0.715 | 3 | 1.335 | | 10 | 1.178 | 10 | 7.401 | 8 |
|  | *Ach* | 1.602 | 7 | 1.676 | 0.896 | 5 | 1.087 | | 8 | 0.711 | 7 | 6.654 | 6 |
|  | *Act* | 1.549 | 6 | 2.057 | 0.951 | 10 | 1.002 | | 6 | 0.676 | 6 | 6.817 | 7 |
|  | *Cht2* | 2.737 | 12 | 1.706 | 0.486 | 6 | 1.773 | | 12 | 1.724 | 12 | 10.091 | 11 |
|  | *EF1α* | 1.379 | 1 | 1.866 | 0.970 | 7 | 0.799 | | 3 | 0.452 | 2 | 2.546 | 3 |
|  | *GAPDH* | 1.633 | 8 | 1.972 | 0.904 | 9 | 1.042 | | 7 | 0.788 | 8 | 7.969 | 9 |
|  | *His* | 2.686 | 11 | 2.560 | 0.691 | 12 | 1.578 | | 11 | 1.673 | 11 | 11.242 | 12 |
|  | *Hsp70* | 1.385 | 2 | 1.660 | 0.948 | 4 | 0.747 | | 1 | 0.431 | 1 | 1.682 | 1 |
|  | *RP49* | 1.505 | 5 | 1.525 | 0.915 | 2 | 0.906 | | 4 | 0.596 | 5 | 3.761 | 4 |
|  | *RPL32* | 1.413 | 3 | 1.398 | 0.896 | 1 | 0.747 | | 1 | 0.456 | 3 | 1.732 | 2 |
|  | *SDH* | 1.680 | 9 | 2.149 | 0.954 | 11 | 1.140 | | 9 | 0.820 | 9 | 9.463 | 10 |
|  | *Tub* | 1.473 | 4 | 1.948 | 0.955 | 8 | 0.969 | | 5 | 0.563 | 4 | 5.030 | 5 |

StdDev, standard deviation; SV, stability value; SD, standard deviation of C_t_ value; r, Pearson correlation coefficient; **p*≤0.001. *p*-value associated with the Pearson coefficient of correlation.
